# Editorial: Advanced Cell Culture Technologies to Boost Cell-Based Therapies

**DOI:** 10.3389/fbioe.2021.727298

**Published:** 2021-07-19

**Authors:** Dominik Egger, Aldo R. Boccaccini, Diego Correa, Cornelia Kasper, Fergal J. O’Brien

**Affiliations:** ^1^ Institute of Cell and Tissue Culture Technologies, Department of Biotechnology, University of Natural Resources and Life Sciences, Vienna, Austria; ^2^ Institute of Biomaterials, Department of Materials Science and Engineering, University of Erlangen Nuremberg, Erlangen, Germany; ^3^ Diabetes Research Institute & Cell Transplantation Center, University of Miami, Miller School of Medicine, Miami, FL, United States; ^4^ Tissue Engineering Research Group (TERG) & AMBER Centre Department of Anatomy & Regenerative Medicine, Royal College of Surgeons in Ireland (RCSI), Dublin, Ireland

**Keywords:** 3D cell culture, physiological conditions, cell-based therapy, therapeutic potential, stem cells

Approved cells and cellular products for cell-based therapy (CBT) applications carry a huge promise for the treatment of a broad variety of diseases and several (stem) cell therapies. However, the therapeutic potential of cells is not fully exploited at present. On the one hand, outdated culture conditions are still used during *in vitro* cultivation. On the other hand, technological hurdles block the way to efficient and safe cell-based therapy products. This Research Topic gathered articles about recent advances in cell culture technologies to increase the therapeutic properties of cells or the manufacturing processes for CBT.

Oxygen has an outstanding role as a cell culture parameter as it is involved in numerous cellular processes and the generation of energy. Tse et al. highlighted in their review article the importance of physiological oxygenation in 3D cultures while keeping anoxic regions at a minimum. With finite element modeling the authors demonstrated the degree of anoxic tissue in standard and gas-permeable plates. Further methods such as transwell plates, microfluidic or bioreactor systems to improve oxygenation in 3D cultures are presented. To monitor oxygen gradients occurring inside spheroids, Schmitz et al. reported the development of a modified hypoxia reporter MSC cell line with a genetic sensor for hypoxic conditions. Using this reporter cell line, the authors demonstrated that the method for producing MSC spheroids and cell number per spheroid play a crucial role in the onset of hypoxia in MSC spheroids. The cell line represents a reliable tool for monitoring hypoxic conditions inside spheroids which may be used to increase comparability between different spheroid production systems.

Besides oxygen, also the culture format itself heavily impacts cellular functionalities. Harnessing 3D spheroid culture for MSCs is known to increase therapeutically relevant effects. Kouroupis and Correa summarize in their review article current methods for the generation of MSC spheroids and how spheroid culture increases functionality of MSCs in various therapeutic applications. Thus, recent findings on the anti-inflammatory and therapeutic properties of MSC spheroids in wound healing, osteochondral defects, myocardial infarction, neovascularization/ischemia and liver and kidney diseases are summarized. Findings from this review demonstrate the need to intensify research on and translation of MSC spheroids into clinical applications. Rüger et al. presented an innovative 3D *in vitro* vascular niche model to observe *de novo* vessel formation by vasculogenesis. The model consists of blood-derived progenitor cells, mature immune cells and MSCs, but not mature ECs, providing an environment which led to maturation of ECs through cellular, extracellular and paracrine cross-talk. This model opens up a new possibility for the *in vitro* engineering of autologous blood vessels. Babajani et al. summarized in their review article recent findings in using MSCs as drug delivery system for chemotherapeutic drugs while focusing on possible adverse effects of chemotherapeutics on MSCs and the efficacy of drug loading and releasing.

For applications in cell-based therapies, MSCs and other cell types need to be amplified or expanded to high numbers while keeping their functionality and critical stem cell properties. Thus, advanced methods for the safe expansion of these cells are required. In this context, Nath et al. reviewed the state of the art on technologies for manufacturing of CBTs and point out the current limitations and bottlenecks. They conclude that automated bioreactors are a key technology for providing CBTs in the future. Furthermore, efforts should be made to perform all steps of the production process (genetic modification, expansion, differentiation) in one integrated bioreactor to provide cost-effective solutions. The use of platelet lysate (PL) as serum alternative has become central in manufacturing of CBTs. Kirsch et al. demonstrated that human PL as media supplement is superior to fetal bovine serum (FBS) and human serum for the expansion and differentiation of MSCs, both in 2D and 3D. The cells exhibited enhanced proliferation and differentiation in 2D culture, compared to FBS or human serum. Furthermore, human PL increased cell spreading and proliferation in gelatin-methacryloyl hydrogels. This study underlines the suitability of PL for the culture of MSCs and proof its advantages also for 3D applications. While PL is already available for culture of human cells, there is a lack of standardized processes for the production of equine PL. Hagen et al. presented the production of equine PL for the use as serum alternative in equine MSC culture. If used at the same concentration, equine PL supports MSC expansion as well as adipogenic and osteogenic differentiation comparable to FBS. As cellular therapies experience a notable shift towards the use of small extracellular vesicles (sEVs), there is an urgent need for chemically-defined and xeno-free culture media specialized for the production of EVs. Interestingly, the study of Figueroa-Valdés et al. presented a suitable xeno-free, blood-free and chemically-defined media for the production of MSC-sEVs. An increased MSC-sEV secretion and characteristic expression pattern of sEV markers was observed while retaining the parental cell’s stem cell phenotype. Consequently, this medium could enable the large-scale manufacturing of MSC-sEVs under regulatory compliant conditions. To achieve reasonable cell numbers for cell-therapies, advanced processes for the dynamic expansion of MSCs are still required. Van Beylen et al. present an approach to screen for suitable microcarriers for the expansion of MSCs with the later aim for bone formation in an *in vivo* mouse model. They found a microcarrier that supported MSC expansion while keeping potency and functionality regarding *in vivo* bone formation. The microcarrier-based expansion process could be used for the large-scale production of MSCs with subsequent *in vivo* bone formation.

In summary, this article collection provides a comprehensive review of the state of the art in advanced cell culture technologies to boost cell-based therapies which we believe will be of significant interest to the journal readership.

